# Low-dose PTCy plus low-dose ATG as GVHD prophylaxis after UD-PBSCT for hematologic malignancies: a prospective, multicenter, randomized controlled trial

**DOI:** 10.1038/s41408-022-00771-w

**Published:** 2023-01-11

**Authors:** Yingling Zu, Ruirui Gui, Zhen Li, Juan Wang, Yanli Zhang, Fengkuan Yu, Huifang Zhao, Xinrong Zhan, Zhongliang Wang, Pengtao Xing, Xianjing Wang, Huili Wang, Yongping Song, Jian Zhou

**Affiliations:** 1 Department of Hematology, Affiliated Cancer Hospital of Zhengzhou University and Henan Cancer Hospital, Zhengzhou, 450000 Henan China; 2 Department of Hematology, Central Hospital of Xinxiang, Xinxiang, 453000 Henan China; 3 Department of Hematology, The Third People’s Hospital of Zhengzhou, Zhengzhou, 450000 Henan China; 4 Department of Hematology, the First Affiliated Hospital of Zhengzhou University, Zhengzhou, 450052 Henan China

**Keywords:** Haematopoietic stem cells, Immunosurveillance

Dear Editor,

For unrelated donor hematopoietic stem cell transplantation (UD-HSCT), graft-versus-host disease (GVHD) and non-relapse mortality (NRM) remain the main factors in success [1]. It is undeniable that anti-thymocyte globulin (ATG) is the standard of care of GVHD prophylaxis for patients undergoing UD-HSCT, due to preventing acute GVHD (aGVHD) and chronic GVHD (cGVHD) effectively [2]. However, the use of ATG is frequently accompanied by high rates of GVHD and infections, especially cytomegalovirus (CMV) and Epstein-Barr virus (EBV) infection [3], affecting the long-term survival of the patients. Post-transplant cyclophosphamide (PTCy) has been a milestone in haploidentical HSCT (haplo-HSCT) by mitigating bidirectional alloreactivity and promoting graft tolerance to mitigate the risk of GVHD [4] and become prevalent in UD-HSCT [5, 6]. It is worth mentioning that the superiority of PTCy has been predominantly reflected in bone marrow (BM) as the source of stem cells and peripheral blood stem cell (PBSC) graft displayed high risk of GVHD compared to BM graft [7].

Consequently, the joint regimen of PTCy and ATG at different dose was progressively implemented in haplo-HSCT, and extended to UD-HSCT to improved long-term outcomes[8, 9]. The combination of low-dose ATG (5 mg/kg) and low-dose PTCy (one dose, 50 mg/kg) as GVHD prophylaxis reduced the risk of GVHD in haplo-HSCT and matched unrelated donor peripheral blood stem cell transplantion (MUD-PBSCT) [8, 10]. In addition, the joint use of low-dose PTCy (14.5 mg/kg on days 3 and 4) plus standard-dose ATG as well as low-dose ATG (4.5 mg/kg) plus standard-dose PTCy (50 mg/kg on days 3 and 4) demonstrated significant improvements in the rates of GVHD and NRM in haplo-HSCT and UD-HSCT [9, 11].

Although those joint strategies are effective as GVHD prophylaxis, there is paucity of comparative data between low-dose PTCy-ATG versus quadruplet ATG based regimen in UD-PBSCT. We initiated a prospective, multicenter, randomized controlled clinical trial to assess the efficacy of the novel regimen for GVHD prophylaxis based on low-dose rabbit ATG (6 mg/kg, Sanofi-Aventis) and low-dose PTCy (20 mg/kg on days 3 and 4), cyclosporin A (CsA) and mycophenolate mofetil (MMF) initiating on day +5 in UD-PBSCT (ChiCTR2200056979). The patients of control cohort were performed with quadruplet ATG based regimen, ATG 2.0 mg/kg/day on days −5 to −1 and short-course methotrexate (MTX) 10 mg/m^2^ on day +1 followed by 7 mg/m^2^ on days +3, +6, and +11, with CsA and MMF on day −1.

From January 2019 to March 2022, a total of 162 patients with hematological malignancies at ages 14–62- years from 3 institutions were eligible in the study and a total of 160 patients were enrolled. The patients were randomly one-to-one assigned to the low-dose PTCy-ATG and the quadruplet ATG cohorts (Supplementary Fig. 1). All patients were eligible for adequate baseline laboratory values. This study had ethical approval from ethical committees of each center and in accordance with the Declaration of Helsinki. All patients provided informed consent prior HSCT. The conditioning regimens for all patients were based on fludarabine 30 mg/m^2^/day on days −6 to −2, busulfan at cumulative dose of 51.2 mg/kg divided into 16 doses on days −5 to −2, cytarabine (Ara-C) 2.0 g/m^2^/day on days −6 to −2. The patients with donor specific antibody-positive (with a median fluorescent intensity ≥ 10,000) were administered 2.5–3 Gy of total body irradiation on day −7. Rituximab at dose of 100 mg on day +5 was given to patients in the low-dose PTCy-ATG cohort as EBV prophylaxis.

There was no difference in the baseline characteristics between both groups except that the patients receiving a graft that was allele mismatches in the low-dose PTCy-ATG cohort were more than those in the quadruplet ATG cohort (48.8% vs. 30.0%, *P* = 0.047) (Supplementary Table 1). The median follow-up time was 457 (44-1376) days in the low-dose PTCy-ATG cohort as compared to 424 (8-1394) days in the quadruplet ATG cohort (*P* = 0.325).

Primary graft failure was documented in two patients in each cohort. One recipient in each of the two groups had mixed chimerisms at day +30 (Table 1). Two patients underwent second salvage allo-HSCT in the low-dose PTCy-ATG cohort. No significant difference in mononuclear cells and CD34^+^ cells was observed in both cohorts. The median times to neutrophil and platelet recovery in cohort low-dose PTCy-ATG were 2 days shorter compared to those in cohort quadruplet ATG (12 days vs. 14 days; *P* = 0.000; and 12 days vs. 14 days; *P* = 0.000, respectively) (Table 1), which inconsistent with that the reconstitution advantage of PBSC might be negated [12]. Accelerated the hematopoietic reconstitution of addition to low-dose ATG in the low-dose PTCy-ATG cohort and delayed engraftment due to use of MTX in the quadruplet ATG cohort probably explained the finding.

**Table 1:** Outcomes and complications of two cohorts.

Variables	PTCy-ATG group(*N* = 80)	Quadruplet ATG group(*N* = 80)	*P* values
Time to ANC recovery (Median,days)	12(10–15)	14(9–31)	<0.001
Time to platelets recovery (Median,days)	12(9–22)	14(9–66)	<0.001
Chimerism at day +30(*n*, %)			
Full donor chimerism	79(98.8)	79(98.8)	1.000
Cumulative incidence GVHD % (95% CI)			
Grade II–IV aGVHD at day +100	23.8(15.1–33.6)	40.0(39.2–50.6)	0.026
Grade III–IV aGVHD at day +100	6.3(2.3–13.0)	17.5(10.1–26.6)	0.029
cGVHD at 2 years	14.7(7.7–23.7)	26.7(17.4–36.8)	0.047
Moderate/Severe cGVHD at 2 years	4.2(1.1–10.7)	11.5(5.6–19.8)	0.073
Cumulative incidence % (95%CI)			
Non-relapse mortality at 2 years	19.5(11.0–29.8)	28.7(18.2–40.1)	0.162
Relapse at 2 years	5.5(1.8–12.6)	3.8(1.0–9.7)	0.668
Disease-free survival at 2 years	75.0(69.3–80.3)	67.4(61.5–73.3)	0.228
Overall survival at 2 years	79.4(74.3–84.5)	68.4(61.6–73.2)	0.055
GVHD and relapse-free survival at 2 years	66.1(60.4–71.8)	49.3(42.9–55.7)	0.032
Cumulative incidence % (95%CI)			
CMV reactivation at day 180	53.6(44.9–66.8)	40.0(29.2–50.6)	0.053
CMV disease at day 180	5.0(1.6–11.4)	2.5(0.5–7.9)	0.407
EBV reactivation at day 180	11.3(5.5–19.3)	68.8(57.2–77.8)	<0.001
Other complications (*n*, %)			
PTLD	1(1.3)	0(0.0)	0.316
Pulmonia	32(40.0)	39(48.8)	0.265
Bacteremia	5(6.3)	9(11.3)	0.263
Hemorrhagic cystitis	34(42.5)	47(58.8)	0.040
TMA	5(6.3)	2(2.5)	0.246
PRES	4(5.0)	3(3.8)	0.699
VOD	2(2.5)	2(2.5)	1.000

*ANC* absolute neutrophil count, *aGVHD* acute graft-versus-host disease, *cGVHD* chronic graft-versus-host disease, *CI* cumulative incidence, *CMV* cytomegalovirus, *EBV* Epstein-Barr virus, *PTLD* posttransplantation lymphoproliferative disorders, *TMA* thrombotic microangiopathy, *PRES* posterior reversible encephalopathy syndrome, *VOD* veno-occlusive disease of the liver.

On competing risk analysis, the 100-day cumulative incidences (CIs) of grade II–IV and III–IV aGVHD in the low-dose PTCy-ATG cohort were significantly lower as compared with those in the quadruplet ATG cohort (23.8% vs. 40.0%, *P* = 0.026; 6.3% vs. 17.5%, *P* = 0.029, respectively) (Table 1) (Fig. 1A; Fig. 1B). The decrease was also noted in 2-year CI of cGVHD compared to quadruplet ATG cohort (14.7% vs. 26.7%, *P* = 0.047) (Fig. 1C). We found no difference in moderate to severe cGVHD at 2 years between the study cohorts (Fig. 1D). Furthermore, an exploratory post-hoc subgroup analysis of grade III–IV aGVHD is depicted in Supplementary Fig. 2, which was not powered to show an effect between two groups. However, it was a trend that HLA mismatched patients preferred to the novel regimen compared to HLA-matched patients in III–IV grade aGVHD end point. HLA-matched degree was the foremost element related to GVHD presence. Previous research showed that the CIs of aGVHD and cGVHD in patients receiving UD-HSCT were comparable with standard PTCy-based regimen or low-dose PTCy-ATG regimen for GVHD prophylaxis [10, 13]. Though those results suggested HLA-matched intensity might not affect the incidences of GVHD under the platforms of PTCy or PTCy-ATG, the results should be interpreted with caution due to limited sample size. In this study, the DQ mismatch in HLA 9/10 mismatched unrelated donor PBSCT was no difference between two cohorts (Supplementary Table 1). Due to limited number of patients, we need to enlarge the sample size to further analyze the effect of different mismatch HLA loci on the GVHD.

**Fig. 1:**
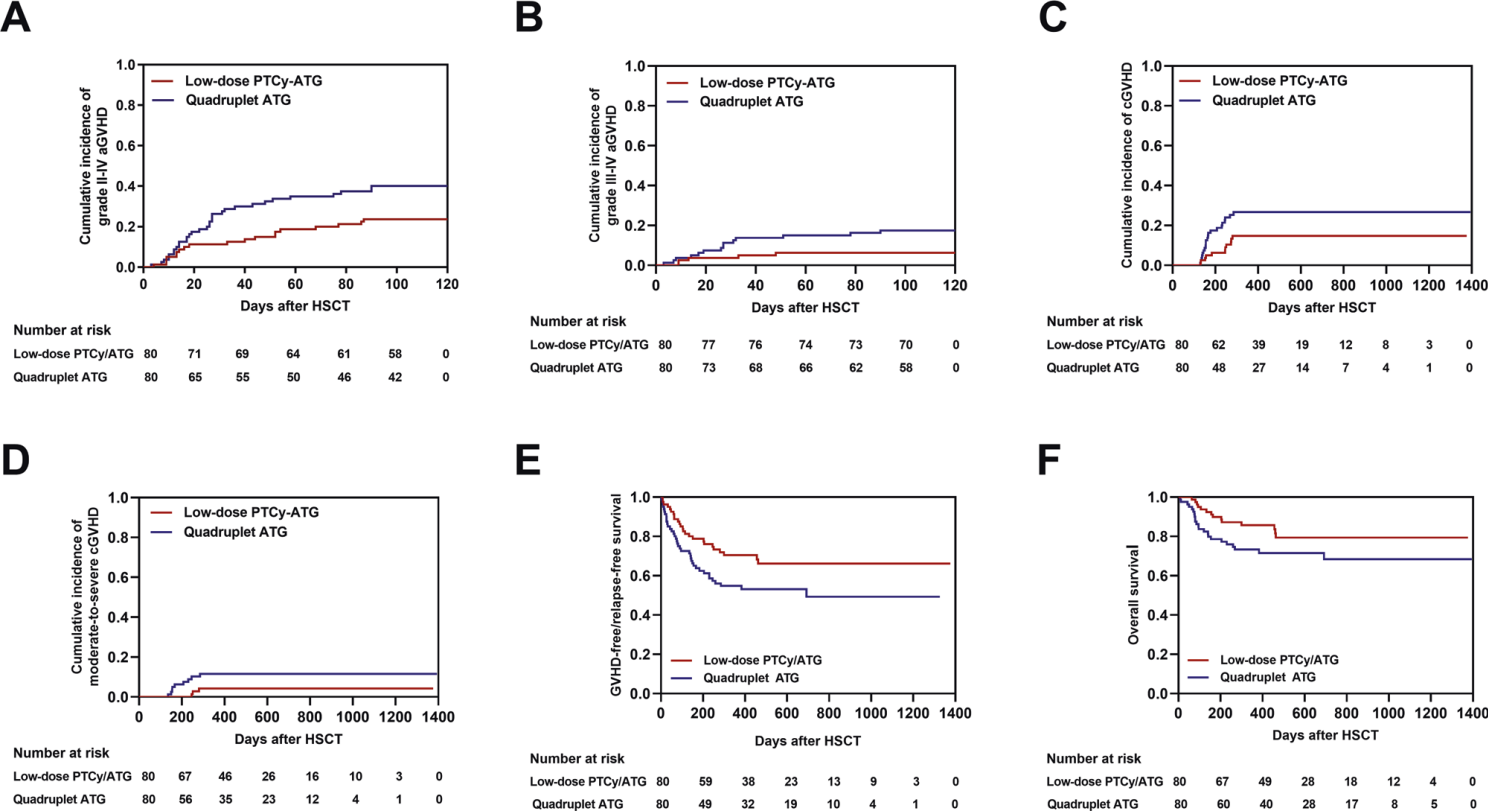
Cumulative incidences (CIs) of graft-versus-host-disease (GVHD) and clinical outcomes between low-dose post-transplant cyclophosphamide (PTCy) combined with low-dose anti-thymocyte globulin (ATG) and quadruplet ATG cohorts. **A** The CI of grade II–IV acute GVHD (aGVHD); **B** The CI of grade III–IV aGVHD; **C** The 2-yaer CI of chronic GVHD (cGVHD); **D** The 2-yaer CI of moderate to severe cGVHD; **E** The 2-yaer CI of GVHD-free, relapse-free survival (GRFS); **F** The 2-yaer probability of overall survival (OS).

Worth mentioning is that the CI of CMV reactivation at day 180 had a trend to increase for patients with low-dose PTCy-ATG, even if there was no significant difference (53.6% vs. 40.0%, *P* = 0.053) (Table 1). A recent Center for International Blood and Marrow Transplant Research study demonstrated that PTCy doubled the risk of CMV infection for CMV seropositive recipients. PTCy could give priority to sparing and recover suppressive regulatory T-cells by selectively impairing allo-reactive T-cells [14], which may affecting cellular immunity against CMV. EBV reactivation is another common complication of infection. Our results showed that the risk of EBV reactivation was significantly reduced in the low-dose PTCy-ATG cohort (11.3% vs. 68.8%, *P* < 0.001), which may be due to the administration of prophylactic rituximab, and it was a small flaw due to mismatch on rituximab between the two groups. However, the dose and timing of rituximab as given is mostly empirical. What is more, it is controversial whether rituximab administration early has an impact on the occurrence of GVHD [15]. In other complications, no significant difference was observed in the incidence of pulmonia, bacteremia, transplant-associated thrombotic microangiopathy (TMA), posterior reversible encephalopathy syndrome and veno-occlusive disease of the liver except that the significant lower was noted in the incidence of hemorrhagic cystitis with BK virus compare to quadruplet ATG cohort (42.5% vs. 58.8%, *P* = 0.040) within the duration of follow-up (Table 1). It was worth mentioning that the rate of TMA was 6.3%. Whether it related to the administration of PTCy deserves further study.

In terms to the outcomes, the 2-yaer CIs of NRM and CIs of relapse (CIR) between two cohorts were comparable (*P* = 0.162; *P* = 0.668, respectively)(Supplementary Fig. 3A, B). Interesting that the CIR did not associated with the decrease in GVHD and NRM. Then, we observed a significantly better GVHD-free/relapse-free survival (GRFS) in the low-dose PTCy-ATG cohort (66.1%) compared with the quadruplet ATG cohort (49.3%) (*P* = 0.032) (Fig. 1E), which indicating the combination of low-dose PTCy and low-dose ATG had the potential to assist patients to gain an improved quality of life. The 2-year probability of disease-free survival was comparable (*P* = 0.228) (Supplementary Fig. 3C). Finally, low-dose PTCy-ATG had a trend to improve OS for patients after UD-PBSCT (79.4% vs. 68.4%; *P* = 0.055) (Fig. 1F), albeit no statistical difference. The causes of death is depicted in Supplementary Table 2.

In conclusion, this is a pilot study to establish a low-dose PTCy and low-dose ATG regimen to effectively prevent GVHD in UD-HSCT. Further studies with high methodological quality, such as further investigations of the optimal dose and schedule of the combination of ATG and PTCy are warranted in the future to verify the feasibility of the strategy.

## Supplementary information

Supplementary data

Supplementary Figure 1

Supplementary Figure 2

Supplementary Figure 3

## Data Availability

The datasets used and/or analyzed during the current study are available from the corresponding author on reasonable request.
