# Supplementary material for: Low-dose PTCy plus low-dose ATG as GVHD prophylaxis after UD-PBSCT for hematologic malignancies: a prospective, multicenter, randomized controlled trial
Source: Blood Cancer J. 2023 Jan 11;13(1):10. doi: 10.1038/s41408-022-00771-w (PMC9834295; doi:10.1038/s41408-022-00771-w)
Supplement: Supplementary file 1 — Supplementary data [file 41408_2022_771_MOESM1_ESM.doc]

Low-dose post-transplant cyclophosphamide plus low-dose anti-thymocyte globulin as GVHD prophylaxis after unrelated donor peripheral blood stem cell transplantation with myeloablative conditioning for hematologic malignancies: a prospective, multicenter, randomized controlled trial

**Materials and methods**

**Patients’ eligibility**

Patients were ineligible if they had active autoimmune disease, uncontrolled active bacterial, viral or fungal infections, severe organ dysfunction, or participated in other clinical research within the last month.

**End points and definitions**

The primary study end point was the cumulative incidence (CI) of grade III–IV acute graft versus host disease (aGVHD). Secondary end points included the engraftment, the CIs of grades II–IV aGVHD and chronic GVHD (cGVHD), the cumulative incidence of relapse (CIR) and non-relapse mortality (NRM), overall survival (OS), disease-free survival (DFS), and GVHD-free/relapse-free survival (GRFS). Neutrophil engraftment was defined as the first of three consecutive days with absolute neutrophil count (ANC) ≥0.5 × 10^9^/L without granulocyte colony stimulating factor (G-CSF), and platelet engraftment was defined as the first of seven consecutive days when platelet count ≥20 × 10^9^/L without platelet transfusion. CR and relapse were defined according to European Society for Blood and Marrow Transplantation (EBMT) criteria [1,2]. Full donor chimerism was defined as ≥ 95% leukocytes of donor origin in bone marrow (BM) and/or peripheral blood samples. GVHD was defined and graded by published criteria [3,4]. OS was defined as the time to death regardless of any cause. DFS was defined as survival with no evidence of relapse or progression after transplant. NRM was defined as death without relapse or progression. GRFS was defined as the first event of grade III–IV aGVHD, cGVHD requiring systemic immunosuppressive treatment, relapse, or death from any cause during follow-up after transplant.

**Sample size and randomization**

The sample size was performed with PASS version 15.0.5 (NCSS Statistical Software). The 100-day CI of grade III–IV aGVHD of quadruplet ATG cohort was 25.0% in the the preliminary research. Calculation of sample size was determined by a reduction from 25.0% to expected 7.0% with a power of 80% and a two-sided test of α = 0.05 type I error. After adjusting for a 10% dropout, a total of 160 participants was required (80 in each group) and the participants were randomly assigned to each arm by a centralized, 24-hour, internet-based randomization system.

**Statistical analyses**

Percentages for categorical variables and continuous variables were reported with median and ranges. Comparisons of groups’ characteristics were performed by Mann-Whitney test for continuous variables and chi-square or Fisher’s exact test for categorical variables, respectively. OS, DFS, and GRFS were calculated using the Kaplan–Meier estimator and compared using the log-rank test. The CIs were estimated aGVHD, cGVHD, relapse and NRM, and comparisons were made by Gray’s test. Relapse was a competing event for NRM and vice versa. Graft failure, relapse, or death other than GVHD were the competing event for GVHD. All *P* values were two-sided and the type I error rate was fixed at 0.05. Statistical analyses were performed with SAS version 9.4 (SAS Institute, Cary, NC) and GraphPad Prism 8.0.2 (GraphPad Software Inc.).

Supplementary Table 1. Patient characteristics and transplant-related parameters

| Variables | PTCy-ATG group(N=80) | Quadruplet ATG  group(N=80) | *P* values |
| --- | --- | --- | --- |
| Median age in years (range)  Recipient sex  Male  Female  Disease type  Acute myeloid leukemia (AML)  Acute lymphoblastic leukemia (ALL)  Myelodysplastic syndromes (MDS)  Lymphoma  Others  HCT-CI  ≥3  <3  Disease risk index  Low/intermediate  High/very high  Disease status before HSCT  CR1  ≥CR2  Not in remission  Minimal residual disease at transplant  Negative  Positive  KPS  < 90  ≥90  Matched HLA loci, n (%)  8/10  9/10  10/10  Donor sex  Male  Female  Donor age (year, median, range)  Donor-recipient pair  Female to female  Female to male  Male to female  Male to male  Blood type matching  Match  Mismatch  HLA 9/10 mismatch loci  DQB1  Median mononuclear cell (range, 10^8^/kg)  Median CD34 + cells (range, 10^6^/kg)  Median follow-up (range, days)  Median follow-up in survivors (range, days) | 30(14-59)  44(55.0%)  36(45.0%)  32(40.0%)  28(35.0%)  12(15.0%)  4(5.0%)  4(5.0%)  14(17.5%)  66(82.5%)  32(40.0%)  48(60.0%)  54(67.5%)  10(12.5%)  16(20.0%)  62(77.5%)  18(22.5%)  20(25.0%)  60(75.0%)  2(2.5%)  37(46.3%)  41(51.3%)  70(87.5%)  10(12.5%)  31(19-48)  4(5.0%)  6(7.5%)  32(40.0%)  38(47.5%)  24(30.0%)  56(70.0%)  2(5.4%)  11.53(1.11-28.14)  6.25 (0.16-18.69)  457(44-1376)  525(183-1376) | 33(14-62)  48(60.0%)  32(40.0%)  40(50.0%)  26(32.5%)  10(12.5%)  2(2.5%)  2(2.5%)  12(15.0%)  68(85.0%)  36(45.0%)  44(55.0%)  58(72.5%)  12(15.0%)  10(12.5%)  68(85.0%)  12(15.0%)  16(20.0%)  64(80.0%)  2(2.5%)  22(27.5%)  56(70.0%)  72(90.0%)  8(10.0%)  32(18-56)  2(2.5%)  6(7.5%)  30(37.5%)  42(52.5%)  22(27.5%)  58(72.5%)  1(4.5%)  11.78(4.93-34.60)  7.08(0.49-16.47)  424 (8-1394)  580(170-1394) | 0.206  0.632  0.647  0.831  0.632  0.425  0.224  0.449  0.047  0.617  0.958  0.818  0.727  1.000  0.631  0.931  0.325  0.836 |

Abbreviations: PTCy, post-transplant cyclophosphamide; ATG, anti-thymocyte globulin; AML, acute myelocytic leukemia; ALL, acute lymphocyte leukemia; MDS, myelodysplastic syndrome; HCT-CI, hematopoietic cell transplantation-comorbidity index; CR1, first complete remission; CR2, second complete remission after relapse; KPS, Kanofsky performance score; HLA, human leukocyte antigen.

Supplementary Table 2 Causes of death

| Cause of death(n, %) | PTCy-ATG group (N=15) | Quadruplet ATG group (N=24) |
| --- | --- | --- |
| Infection other than CMV/EBV  Relapse  GVHD  Organ failure  TMA  VOD | 7(46.7)  1(6.7)  2(13.3)  1(6.7)  3(20.0)  1(6.7) | 9(37.5)  3(12.5)  5(20.8)  4(16.7)  1(4.2)  2(8.3) |

Abbreviations: CMV, [cytomegalovirus](javascript:void(0);); EBV, Epstein-Barr virus;GVHD, graft-versus-host disease; TMA, thrombotic microangiopathy; VOD, veno-occlusive disease of the liver.

Figure Legends

Fig.1 CONSORT flow diagram of participant recruitment. PTCy, post-transplant cyclophosphamide; ATG, anti-thymocyte globulin.

Fig.2 Forest plot summarizing hazard ratios for the low-dose PTCy-ATG cohort versus the quadruplet ATG cohort in the subgroup analyses with a test for interaction. PTCy, post-transplant cyclophosphamide; ATG, anti-thymocyte globulin; HR, hazard ratio; CI, confidence interval; HCT-CI, hematopoietic cell transplantation-comorbidity index; CR, complete remission; HSCT, haematopoietic stem cell transplantation; MRD, Minimal residual disease.

Fig.3 Clinical outcomes between low-dose post-transplant cyclophosphamide (PTCy) combined with low-dose anti-thymocyte globulin (ATG) and quadruplet ATG cohorts. (A) The 2-yaer CI of non-relapse-mortality (NRM); (B) The 2-yaer CI of relapse; (C) The 2-yaer probability of disease-free survival (DFS).

**References**

1. Schuurhuis, G.J., et al., Minimal/measurable residual disease in AML: a consensus document from the European LeukemiaNet MRD Working Party. Blood, 2018. 131(12): p. 1275-1291.

2. Bacigalupo, A., Hematopoietic stem cell transplants after reduced intensity conditioning regimen (RI-HSCT): report of a workshop of the European group for Blood and Marrow Transplantation (EBMT). Bone Marrow Transplant, 2000. 25(8): p. 803-5.

3. Przepiorka D, Weisdorf D, and M. P, Consensus conference on acute GVHD grading. Bone Marrow Transplantation, 1995. 15: p. 825-8.

4. Jagasia, M.H., et al., National Institutes of Health Consensus Development Project on Criteria for Clinical Trials in Chronic Graft-versus-Host Disease: I. The 2014 Diagnosis and Staging Working Group report. Biol Blood Marrow Transplant, 2015. 21(3): p. 389-401.e1.
