# Supplementary figures and images for: Low-dose PTCy plus low-dose ATG as GVHD prophylaxis after UD-PBSCT for hematologic malignancies: a prospective, multicenter, randomized controlled trial
Source: Blood Cancer J. 2023 Jan 11;13(1):10. doi: 10.1038/s41408-022-00771-w (PMC9834295; doi:10.1038/s41408-022-00771-w)

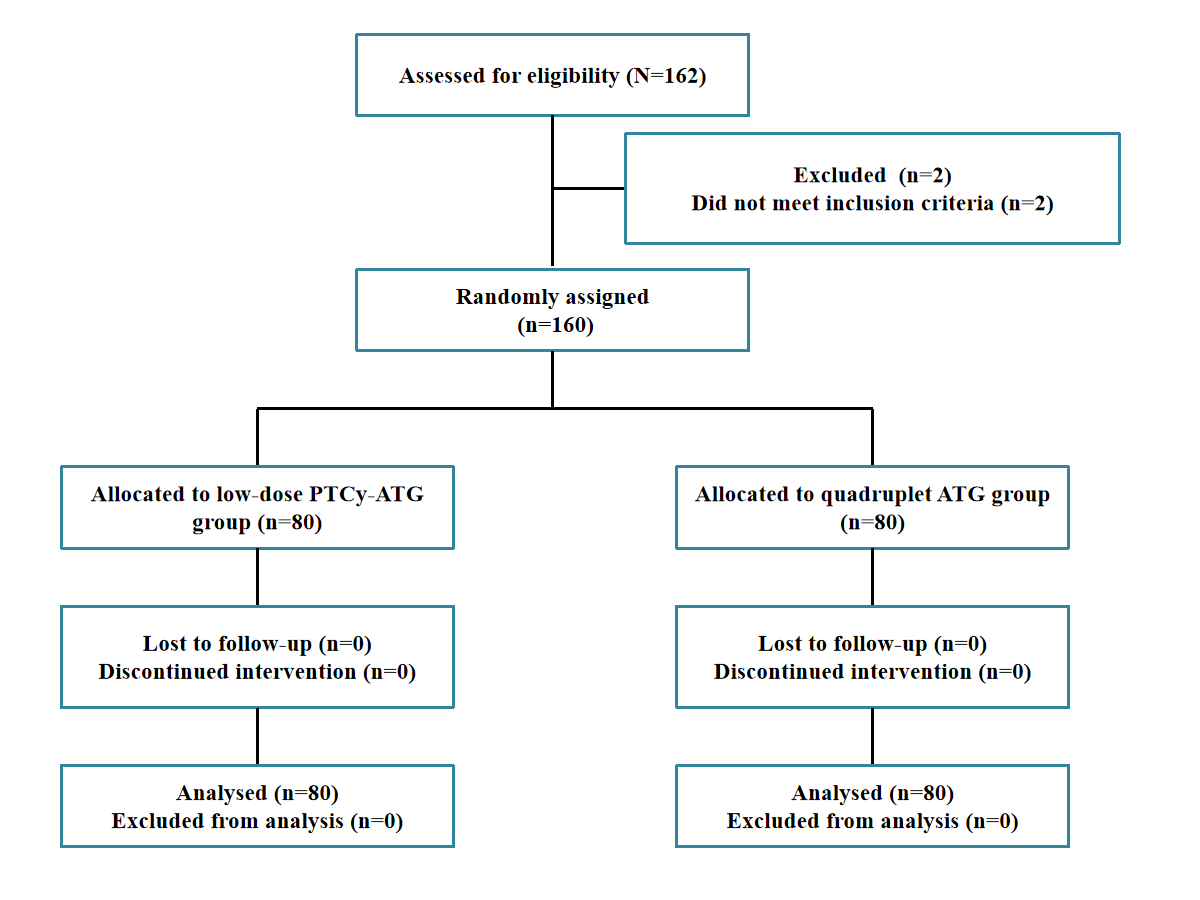

Supplement: Supplementary file 2 — Supplementary Figure 1 [file 41408_2022_771_MOESM2_ESM.tif]

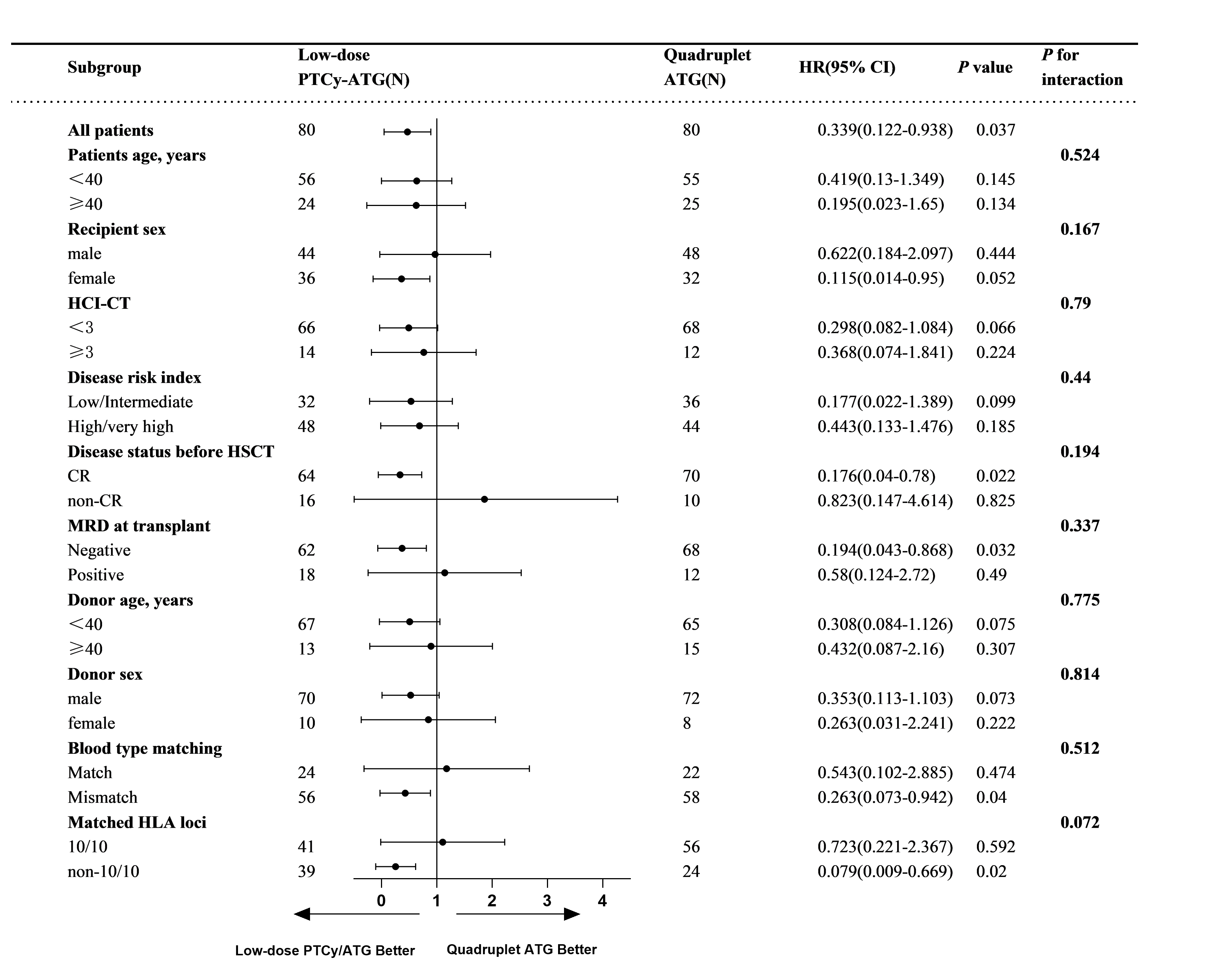

Supplement: Supplementary file 3 — Supplementary Figure 2 [file 41408_2022_771_MOESM3_ESM.tif]

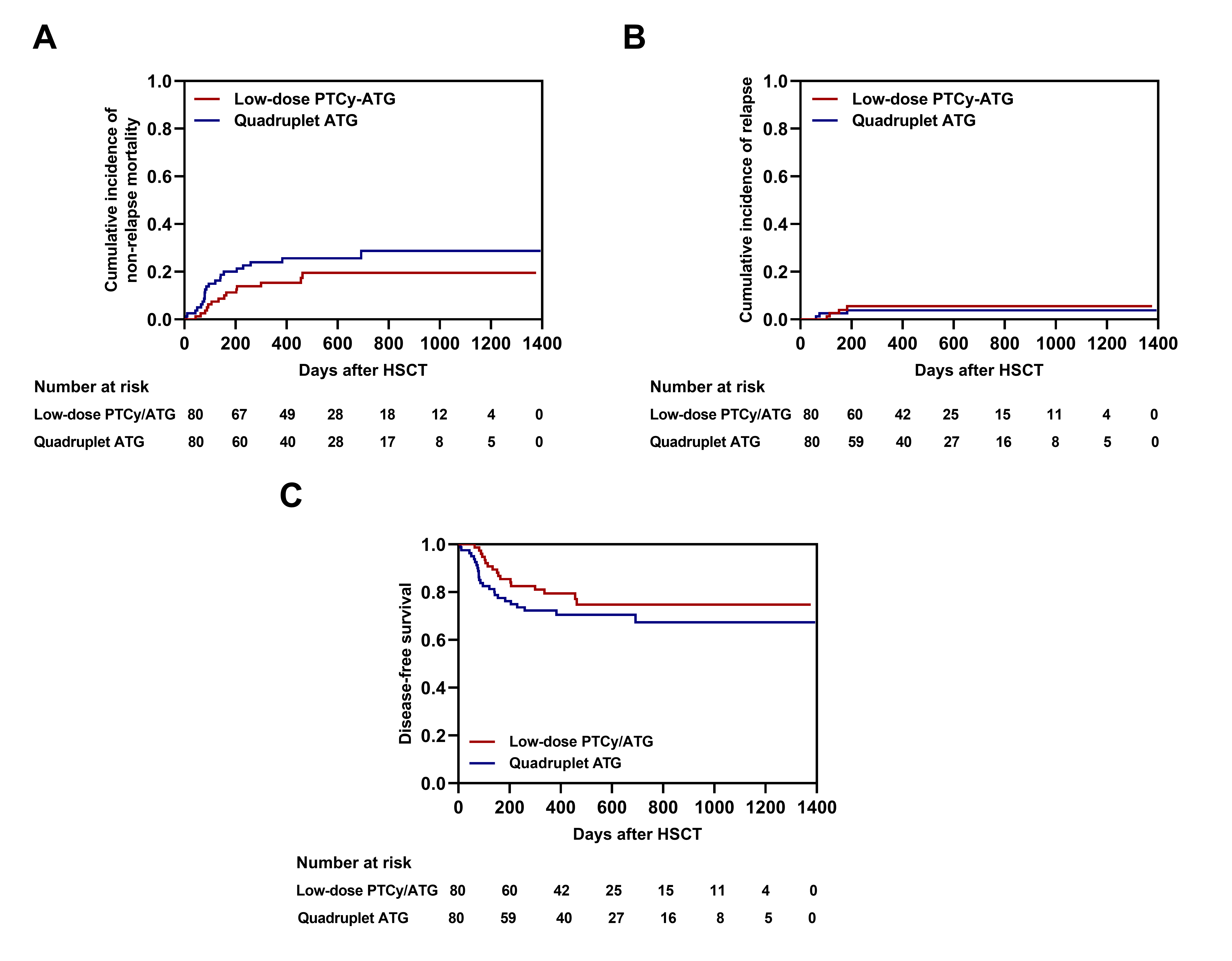

Supplement: Supplementary file 4 — Supplementary Figure 3 [file 41408_2022_771_MOESM4_ESM.tif]
